# Effect of nutmeg essential oil (*Myristica fragrans* Houtt.) on methane production, rumen fermentation, and nutrient digestibility in vitro

**DOI:** 10.1038/s41598-024-52532-3

**Published:** 2024-02-12

**Authors:** Achmad Ezar Abdillah, Dewi Sarah, Aziz Aji Ardian, Muhsin Al Anas, Muhammad Anang Aprianto, Chusnul Hanim, Asih Kurniawati, Muhlisin, Lies Mira Yusiati

**Affiliations:** Laboratory of Nutritional Biochemistry, Faculty of Animal Science, Universitas Gadjah Mada, Sleman Regency, Daerah Istimewa Yogyakarta 55281 Indonesia

**Keywords:** Environmental impact, Biochemistry

## Abstract

The study evaluated the effect of adding of nutmeg (*Myristica fragrans* Houtt.) essential oil **(**NEO) as a feed additive on methane production, rumen fermentation parameters, rumen enzyme activity, and nutrient digestibility in vitro. This study was divided into three treatments based on the level of NEO addition, which included 0 µL/L (T0), 100 µL/L (T1), and 200 µL/L (T2). The feed substrate composition consisted of king grass as forage and concentrate in a 60:40 ratio. Feed fermentation was conducted using the Menke and Steingass gas production and two-step Tilley and Terry in-vitro digestibility technique. The data obtained from the study were analyzed using one-way ANOVA and if there were differences between means, they were further assessed using DMRT. The results showed that T2 treatment significantly decreased (*P* < 0.05) ammonia (NH_3_) levels, total VFA, acetate, propionate, butyrate, and microbial protein (*P* < 0.05). Methane production and the activity of rumen protease enzyme significantly decreased (*P* < 0.05) at T1 and T2 treatment. The T2 treatment significantly reduced (*P* < 0.05) protein digestibility (IVCPD) at 48 h, while IVCPD at 96 h significantly increased (*P* < 0.05). On the other hand, the addition of nutmeg essential oil did not effect the activity of the amylase, carboxymethyl cellulase, and β-glucosidase enzymes, as well as the in-vitro digestibility of dry matter (IVDMD), crude fiber (IVCFD), and organic matter (IVOMD). The conclusion drawn from this study is that the optimum level for NEO is 200 µL/L, which can reduce methane production and increase crude protein digestibility at 96 h without any negative effect on rumen fermentation and nutrient digestibility.

## Introduction

Ruminant farming is one of the primary sources of animal protein with high nutritional values for human consumption, such as meat and milk. However, ruminant farming has a significant negative impact on environmental issues, particularly global warming, due to the production of methane gas (CH_4_)^1^. Methane is the second-most significant greenhouse gas (GHG) after carbon dioxide. The methane gas contributes to the atmosphere of greenhouse gas (GHG), which have potential to contribute in global warming (16–25% of total GHG)^2^. Agriculture stands as the major contributor to global CH_4_ emission resulting from human activities. Approximately 80% of agricultural CH_4_ is produced by livestock systems, with nearly 90% coming from enteric fermentation by ruminants such as cattle and sheep and the remaining 10% coming from animal manure^3^. On the other hand, methane production from enteric fermentation also negatively impacts on the animals. The methane produced in the rumen reflects the loss of feed energy, about 3–12% of digested energy, which should be used for animal growth or milk production^4^. Consequently, this leads to reduced animal productivity^5^. Therefore, ithe development of innovative technologies aimed at reducing methane emission from ruminant is crucial, as it would not only mitigate national greenhouse gas emissions but also increase the available energy for enhancing livestock productivity.

Manipulation the rumen fermentation process through the use of feed additives that stimulate growth and enhance rumen microbial activity can significantly improve feed digestibility and nutrient utilization^6^. In ruminant production, synthetic feed additives, including antibiotics, ionophores, methane inhibitors, and defoaming agents, have been commonly employed to boost meat and milk production, enhance feed efficiency, improve consumption and prevent disease^7^. The use of antibiotics as feed additives has drawn considerable attention and faced restriction in various countries, particularly in Indonesia, due to health concern. Consequently, keeping antibiotic usage to minimum dosage and achieve are sustainable d higher quality production are the aim of livestock industry^8^. As a result, there is a growing need for alternative feed additives to optimize livestock production, such as probiotics and phytobiotics^9^. Extracts of secondary metabolite from plants, such as saponins, tannins, and essential oils have been evaluated for enhancing microbial metabolism in the rumen, increasing efficiency fermentation, and inhibition methanogenesis production^10^.

Nutmeg (*Myristica fragrans* Houtt.) is a prominent commodity in the tropics, with Indonesia being the world’s largest nutmeg producer. Nutmeg is widely utilized in the industry for the production of essential oils through the steam distillation of nutmeg seeds. The process of steam distillation yields several bioactive compounds, including sabinene (21.38%), 4-terpineol (13.92%), and myristicin (13.57%)^11^. In the current study, decreased number of protozoa cells, which is correlated with reduced with methane production, was observed when essential oil such as myristicin^12^ and terpineol^13^ were used as active compounds. However, it's important to note that while essential oils have demonstrated potential in inhibiting methanogenesis in the rumen, some studies have reported negative effects on fiber digestion and fermentation. The extent of these effects depends on the type and dosage of the essential oil and the composition of the diet^14^. Therefore, this study is interested on evaluating nutmeg essential oil, a leading commodity in Indonesia that contains several bioactive compounds like terpineol and myristic. The bioactive compound can potentially reduce methane production, which is a concern in the current ruminant research. As the results, this study carried out to know the best essential oil dose that reduce methane gas production; however, it has no adverse effect on fiber digestion and fermentation.

## Results

### Effect nutmeg essential oil on rumen fermentation parameters

Based on the data in Table 1, the use NEO at 100 µL/mL significantly decreased (*P* < 0.05) the concentration of total VFA. The use up to 200 µL/mL significantly decreased (*P* < 0.05) the concentration of NH_3,_ acetate, propionate, butyrate, and microbial protein. The NEO was no effect (*P* > 0.05) on pH, ratio of acetate and butyrate, and protozoa cells.

**Table 1:** Effect of nutmeg essential oil on rumen fermentation parameters.

Fermentation parameters	Level of nutmeg essential oil (µL/L)		
	0	100 µL	200 µL
pH	6.87 ± 0.01	6.84 ± 0.02	6.86 ± 0.02
NH_3_ (mg/100 mL)	23.34 ± 0.99^a^	22.42 ± 0.58^ab^	20.93 ± 0.19^b^
VFA (mM)			
Acetate (mM)	82.34 ± 1.05^a^	80.50 ± 2.01^a^	73.77 ± 0.93^b^
Propionate (mM)	18.86 ± 1.27^a^	16.85 ± 0.72^ab^	15.56 ± 0.70^b^
Butyrate (mM)	7.70 ± 0.26^a^	7.15 ± 0.53^a^	5.84 ± 0.30^b^
Acetate: Propionate	4.38 ± 0.25	4.78 ± 0.17	4.75 ± 0.27
Total VFA (mM)	113.47 ± 3.47^a^	96.71 ± 2.26^b^	95.19 ± 3.80^b^
Microbial protein (mg/mL)	1.10 ± 0.04^a^	1.07 ± 0.05^a^	0.98 ± 0.01^b^
Protozoa (selx10^3^)	2.72 ± 0.17	2.66 ± 0.13	2.63 ± 0.26

^a, b^ Different superscripts in the same line show a significant effect (*P* < 0.05).

### Effect nutmeg essential oil on methane production

The methane production data is provided in Table 2. Methane production significantly decreased (*P* < 0.05) with using of NEO, starting from 100 µl/L. The reduction in methane was significantly different at 100 µL/L level compared to the control**,** but it was not significantly different from the 200 µL/L level.

**Table 2:** Methane production with the using of nutmeg essential oil.

Parameter	Level of nutmeg essential oil (µL/L)		
	0	100 µL	200 µL
Methane (mL/300mgDM)	14.83 ± 0.48^a^	12.15 ± 0.78^b^	11.65 ± 1.14^b^
Methane/DMD (mL/g)	92.58 ± 2.99^a^	77.12 ± 5.47^b^	75.60 ± 7.67^b^
Methane/OMD (mL/g)	93.58 ± 2.15^a^	78.25 ± 6.66^b^	75.71 ± 8.28^b^

^a, b^ Different superscripts in the same line show a significant effect (*P* < 0.05).

### Effect nutmeg essential oil on rumen enzymes activity

The activity of the protease enzyme significantly decreased (*P* < 0.05) with the use of NEO, starting from 100 µL/mL, while there was no significant difference between the use of 200 µL/L and 100 µL/L. The NEO had no effect (*P* > 0.05) on the activity of the amylase, carboxymethyl cellulase, and β-glucosidase enzymes (Table 3).

**Table 3:** Rumen enzymes activity with the using of nutmeg oil essential oil.

Enzyme activity	Level of nutmeg essential oil (µL/L)		
	0	100 µL	200 µL
Amylase (U/g)^ns^	27.86 ± 2.69	27.83 ± 2.48	26.69 ± 2.91
Carboxymethyl cellulase (U/g)^ns^	1.71 ± 0.09	1.70 ± 0.15	1.51 ± 0.12
β-Glucosidase (U/g)^ns^	55.13 ± 3.44	54.41 ± 1.83	51.40 ± 1.99
Protease (U/g)	63.03 ± 1.47^a^	56.35 ± 2.34^b^	52.12 ± 1.57^b^

^a, b^Different superscripts in the same line show a significant effect (*P* < 0.05).

### Effect Nutmeg essential oil on nutrient digestibility

As shown in Table 4, the use of NEO at the 200 µL/L level significantly decreased (*P* < 0.05) protein digestibility (IVCPD) at 48 h, while IVCPD at 96 h significantly increased (*P* < 0.05). The NEO had no effect (*P* > 0.05) on in-vitro digestibility of crude fiber (IVCFD), organic matter (IVOMD), and dry matter (IVDMD) during 48 and 96 h incubation.

**Table 4:** In vitro digestibility of feed nutrients with the using of nutmeg essential oil.

Nutrient digestibility	Level of nutmeg essential oil (µL/L)		
	0	100	200
Crude protein			
48 h	46.68 ± 0.32^a^	45.39 ± 0.57^ab^	44.24 ± 0.7^b^
96 h	60.95 ± 0.57^b^	62.09 ± 0.47^ab^	63.54 ± 1.06^a^
Crude fiber			
48 h	38.94 ± 0.29	38.59 ± 1.05	37.40 ± 0.89
96 h	47.04 ± 1.38	48.35 ± 0.89	49.30 ± 0.51
Organic matter			
48 h	53.00 ± 1.53	52.25 ± 1.90	50.70 ± 0.89
96 h	50.55 ± 0.54	51.10 ± 0.26	53.20 ± 1.73
Dry matter			
48 h	44.33 ± 2.04	43.20 ± 2.21	42.67 ± 2.23
96 h	52.53 ± 1.32	53.13 ± 1.32	55.13 ± 0.57

^a, b^ Different superscripts in the same line show a significant effect (*P* < 0.05).

## Discussion

Ammonia is produced in the rumen due to the degradation of feed protein by rumen microbes, serving as a source for synthesizing amino acids and microbial cells^15^. The content of rumen ammonia (NH_3_) reflects the degradation activity of feed protein and endogenous protein by rumen microbes, governed by the N balance mechanism in the livestock’s body^10^. Elevated levels of ammonia levels result from increased amino acid deamination, primarily driver by HAP (Hyper Ammonia Producer) bacteria. Excessive ammonia production in the rumen that is not utilized by livestock is excreted through urine in the form of urea^5^. In our current study, the use of NEO at 200 µL/L level reduced ammonia concentration by 10.33% compared to the control. Similarly, rosemary essential oil at 250 µL decreased rumen ammonia concentration by 44.88% compared to the control^16^. These reductions are attributed to the active compounds in essential oils that have reactivity against Hyper Ammonia Producer (HAP) bacteria, specifically phenolic compounds^5^. Essential oils also exhibit anti-microbial activity, reducing HAP bacteria responsible for excessive ammonia production in the rumen and disrupting the activity of *Prevotella* bacteria with proteolytic functions^17^. The optimal range for rumen ammonia concentration for microbial protein synthesis is typically between 5.6 and 10.0 mg/100 mL when energy available in the rumen^18^. In our study, the ammonia concentration is approximately 20.0 mg/100 ml (Table 1), which suggests an optimal concentration for microbial protein synthesis.

Nutmeg essential oil at a concentration of 200 µL/mL reduced total VFA production by 15.75% compared to the control. The decrease in total VFA in the rumen was influenced by the active compounds in NEO and the doses used in the treatment. Similarly, the use of tea tree essential oil, starting from 125 µL/mL, with active compounds such as terpinen-4-ol, γ-terpinene, α-pinene, cymene, α-terpineol, and terpinolene, resulted in a decrease in total VFA^15^. The chemical composition of essential oils, their dosage, and the composition of feed ration can all impact total VFA production in the rumen. The composition of the active compounds of essential oils varies depending on the plant of origin^17^.

Essential oils from various plants, when used at high doses, tend to reduce total VFA production, reflecting a decrease in feed degradation. This is particularly notable in microbes that ferment carbohydrates in the rumen to produce VFAs, which are then used by the animal host as the main energy source^19^. The antibacterial effect of essential oils, such as terpenoid and phenylpropanoid compounds, are attributed to heir hydrophobic and cyclic hydrocarbons. These compounds interact with bacterial cell and the lipid layer, leading to cell membrane leakage and cell death^20^. The typical concentration of VFAs required for microbial protein synthesis falls within the range of 70 to 150 mM^5^. In this study, VFA concentrations ranged from 95.19 to 113.47 mM, which falls within the normal range for microbial protein synthesis.

In the current study, the concentration of microbial protein decreased by 10.91% compared to the control when NEO was added at 200 µL/mL. This reduction in microbial concentration was associated with a decrease in NH_3_ and total VFA, as shown in Table 1. Microbial protein is a product of the fermentation of feed in the rumen, serving as a crucial protein source for ruminants. The substrates involved in microbial proteins synthesis include ammonia as a nitrogen source and VFA as an energy source. Rumen microbes utilize ammonia in the synthesis of microbial protein^21^. The availability of feed nitrogen plays a pivotal role in the formation of microbial protein, with ammonia originating from breakdown of feed protein, feed NPN, or ammonium salts^5^. Furthermore, the synthesis of microbial protein is influenced by the supply of sufficient quantities and types of carbohydrates (CHO) as an as an energy source for peptide bonds formation. Readily fermentable CHO, such as starch or sugars, are more effective in promoting microbial growth than other CHO sources, like cellulose^22^.

The results of this study demonstrated that NEO reduced methane production in rumen fermentation when used at 100 and 200 µL/mL. In line with these findings, the use of oregano essential oil containing eugenol, starting at 13 µL/L, resulted in a decrease in methane production. The reduction in methane where the decreased in methane was proportional to the increased levels of addition^23^. Others research reported that fennel essential oil, when administered at doses of 200 and 400 µL/L, led to a substantial reduction in methane production of 27.03% and 47.75%^24^. Changes in methane production can be attributed to decrease in VFA production, particularly in the proportion of acetic acid, as indicated in Table 1. Acetic acid production generates hydrogen gas (H_2_), which serves a substrate in methanogenesis reactions. A reduction in acetic acid formation reduces the substrate available for methane production^25^. The proportion of propionic acid also decreased with each level of NEO (Table 1). Similarly, the use of *Coleus amboinicus* L., containing the active compound carvacrol, reduced propionic acid production^26^. This decrease can be attributed to a decrease in the substrate for propionic production, which is hydrogen (H_2_), and can be associated with acetic, butyric and protozoa levels (Table 1). Hydrogen (H_2_) is produced during acetic and butyric formation. Additionally, protozoa are the one microbe as the primary source of H_2_ production in the rumen. Consequently, the decreased in acetic, butyric and protozoa levels results in reduced substrate availability for propionic acid formation^5^. 

Based of this study, the activity of protease enzyme significantly decreased with the addition of NEO at 100 and 200 µL/mL by 10.59% and 17.30% compared to the the control. In a previous study, it was reported that the addition of galangal essential oil, starting from 30 µL/300 mg (DM feed) reduced protease enzyme activity. This study indicated that the addition of NEO decreases the protease enzyme activity, potentially leading to reduced digestibility of crude protein and ammonia concentration in the rumen. The decrease in protease enzyme activity is influenced by active compounds in NEO, such as phenols.The mechanism involves the hydroxyl group of phenolic compounds binding to enzyme protein. Phenols are sensitive to the Gram-negative bacteria, including those from the *Prevotella* genus, R. *Amylophilus,* which are an amylolytic and proteolytic bacteria^27^. The inhibition of bacterial protease colonization in the rumen by essential oils is indicated by the decrease in protease activity^28^. This study also reports that amylase, CMC-ase, and β-glucosidase enzyme activities were not influenced by the addition of NEO. In line with this finding, the addition of blend essential oil, including clove bud oleoresin and lemongrass oil at 0%, 0.75%, and 1.5%, had no significant effect on amylase, CMC-ase, and β-glucosidase enzyme activities^29^. Enzymes catalyze the hydrolysis of proteins, fats, and carbohydrates into simpler forms. The measured of digestive enzyme activity can describe the ability of feed nutrient utilization^30^. Nutmeg essential oil contains terpenes that inhibit the breakdown of cellulose. Terpenes are known to inhibit the growth of rumen microorganisms, which are important for cellulose breakdown of^31^. The use of NEO up to 200 µL/mL in this study was found to be safe and did not interfere with enzyme activity in the rumen.

Nutmeg essential oil at concentration of 200 µL/mL decreased in vitro crude protein digestibility (IVCPD) in the rumen (48 h) by 4.27% compared to the control. Similarly, a. previous study reported a decrease of IVCPD in the rumen due to by the addition of cinnamon^32^ and lemongrass^33^ essential oil. The reduction in protein degradation in the rumen, associated with essential oils, is attributed to their selective antimicrobial action against rumen microorganisms, especially proteolytic bacteria, leading to decrease in the activity of rumen protease enzymes^34^. The addition of 200µL/L of NEO reduced the activity of protease enzymes in the rumen (Table 3). The action mechanism of essential oils inhibits bacterial hyperammonemia by phenolic compounds that can bind to the amino groups of proteins, thereby reducing protein deamination in the rumen^28^. This study also reports a reduction in ammonia concentration, which is correlated with protease enzyme activity (Table 1). The decreased in IVCPD observed in this study is also attributed to a reduction in the abundance of proteolytic bacteria in the rumen. Protease enzyme is secreted by proteolytic bacteria to hydrolyze feed proteins entering the rumen into amino acids, which are subsequently deaminated into ammonia^35^. The use of NEO as a source of phenol is also likely inhibits the growth of proteolytic bacteria, thereby reducing protease activity in rumen fluid. The utilization of a blend essential oils known as BEO, consisting of thymol, eugenol, vanillin and limonene of 1000 mg/Kg of feed, inhibited the growth and activity of several proteolytic bacteria (*Clostridium sticklandii, Prevotella and Peptostreptococcus anaerobius*), which are hyperammonia-producing bacteria. (HAP)^36^. Among these bacteria, the *Prevotella ruminicola* strain exhibited the highest protease activity in the rumen. The bioactive component of NEO have a toxic effect on proteolytic bacteria, and synergistic action of nutmeg components, such as phenols and monoterpenes, contributes to their antibacterial effect against the abundance of proteolytic bacteria in the rumen^37^. Other studies have also reported that the addition of eucalyptus and rosemary EO at a dose of 60 µL each, containing main components like cineol and phenolic compounds, significantly reduced total *Prevotella* compared to control samples.

Proteins are broken down by rumen microbes into peptides and amino acids, which serve as intermediate products. Some of these amino acids are further metabolized by rumen microbes into ammonia or used for synthesis microbial protein. Simultaneously, undigested protein in the rumen proceeds the abomasum and the intestinal tract where it is digest by by the protease enzymes of the digestive tract, absorbed, and utilized for metabolism. The decrease in crude protein digestibility in the rumen is advantageous, as it enhances the efficiency of feed protein absorption in the animal host’s intestinal tract, ultimately increasing livestock productivity^24^. In the current study, the addition of NEO at 200µL/L significantly decreased the crude protein digestibility at 48 h or rumen phase by 5.22% compared to the control, while maoicrude protein digestibility at 96 h increased by 4.24% compared to the control. The decrease in the rumen phase can be attributed to the phenol compound in the NEO, which inhibit proteolytic microbial activity, thereby reducing the activity of protease enzyme in the rumen. Essential oils contain functional group OH (phenol) with reactive properties towards macromolecules like proteins through ester bond. These ester bond form due to the presence of hydroxyl group (COOH) on the amino acid, allowing it to bond with the hydroxyl group (OH) of phenol. These bonds are difficult to hydrolyze, thus reducing the activity of the protease enzyme^38^. Bioactive compounds found in the essential oils contain phenol functional groups capable of forming bonds with protein to escape rumen degradation, which can be optimally degraded in the post-rumen^38^.

## Materials and methods

Animal procedure performed in this study was registered with the Research Ethics Committee at the Directorate of Research, Universitas Gadjah Mada. All experimental procedures were performed in accordance with the approved protocols, relevant guidelines and regulations. The study was carried out in compliance with the Animal Research with: Reporting of In Vivo Experiments (ARRIVE) guidelines.

### Substrate and research design

The substrate used in this study was a complete feed comprising of king grass (*Pennisetum purpureum*) as forage and a concentrate mixture of several ingredients, including soybean hulls, peanut hulls, rice hulls, wheat bran, rice bran, palm meal, copra meal, corn, cassava peels, cassava flour, cassava cake, corn gluten feed, salt, minerals, and molasses. The mixture was prepared with a ratio of 60:40. The Nutmeg essential oils (NEO) was obtained from Nutmeg Essential Oil Industry located in Ungaran, Semarang, Central Java, Indonesia. The bioactive compounds of NEO were analyzed using gas chromatography–mass spectrometry (GC–MS, BP20, Shimadzu, Japan) following the method outlined reference^39^. The concentration levels in this study is based in bioactive compound of NEO are presented in Table 5. Various amount of NEO was then diluted in acetone to achieve three concentrations: 0 µL/L (T1), 100µL/L (T2), and 200µL/L (T3). These diluted NEO solutions were added to the buffered inoculums in each tube. Acetone was added to the treatment control (without essential oil) to standardize all treatments and mitigating the potential effects of acetone addition on the studied parameters.

**Table 5:** Bioactive compound of nutmeg essential.

Chemical compound	Percentage (%)
1,3-Benzodioxole	25.735
1,3-Cyclohexadiene	20.353
Benzene	12.378
α-Pinene	9.548
β-Pinene	6.006
Linalool	5.299
Cymene	4.650
Terpineol-4	3.885
Pulegone	2.330
Eugenol	2.239
β-Myrcene	2.224
Carene	2.036
α-Asarone	1.820
γ-Terpinene	1.496

### Proximate analysis

The forage was cut into pieces measuring 2 to 3 cm, and then dried in an oven 5 °C for three days. The concentrate, on the other hand, was dried overnight to determine its dry matter (DM). Subsequently, the feed ingredients were ground to pass a 1 mm screen. The ground samples were then analyzed to determine their organic matter (OM), crude protein (CP), and crude fiber (CF)^40^. The chemical composition of the feed materials used in this study is presented in Table 6.

**Table 6:** The chemical composition of the feed materials.

Feed material	Chemical composition			
	DM (%)	OM (%)	CP (%)	CF (%)
King grass	91.52	87.79	7.15	30.74
Concentrate	90.63	85.20	11.58	17.80

### Preparation of rumen fluid and buffered inoculums

Rumen fluids and buffered inoculums were prepared for gas production, fermentation parameters, and nutrient degradability analysis^41,42^. A number of 1.5 L of rumen fluid was collected from the rumen-fistulated female Bali cattle with an average liveweight of 452 ± 18 kg. These cattle had been fed a diet consisting of a 40:60 ratio of king grass and wheat pollard for one week as part of feed adaptation process. Rumen fluid samples were collected before the morning feeding, meticulously filtered through double layers of cheesecloth, and then transferred into pre-warmed thermos bottles at 39 °C. Subsequently, the rumen fluids were transported to the laboratory within 30 min. The buffered inoculums were created by combining rumen fluids and buffer solution with ratio 1:2 (v/v) for gas production analysis and 1:4 (v/v) for nutrient digestibility analysis under a continuous flux of CO_2_.

### In vitro gas production

Three hundred milligrams of substrate were weighed and placed in 100 mL glass syringe (Fortuna® model, Germany) for gas production analysis. The syringe was placed on a reciprocal shaker in a fan-driven incubator and pre warmed to 39 °C for overnight. Each glass syringe filled with 30 mL of buffered inoculum and incubated for 48 h in an incubator at 39 °C. At the end of incubations period, gas production was recorded and collected. Methane content was determined using a gas chromatograph (7890A GC, Agilent Technologies, Santa Clara, CA, USA). Fermentation products were filtered to separate substrate and liquid samples. The liquid samples were tested for pH using a HI 2210 pH meter (Hanna Instruments, Inc., USA). Another 500 µL liquid samples was fixed by the addition of 400 µL formaldehyde-saline solution (37% [v/v] formaldehyde and 0.9% [w/v] NaCl), then homogenized and determined cell count method described by^43^. Others liquid samples were centrifuged (3000 g, 10 min) to obtain the supernatant. Four hundred microliters of supernatant samples were mixed with 200 µL of 1% H_2_SO_4_ (wt/vol) to determine ammonia–nitrogen (NH_3_–N) concentration using the phenol–sodium hypochlorite colorimetric method described by^44^. Another 1 mL supernatant sample was added with 0.2 mL 25% metaphosphoric acid solution, then homogenized using a vortex, and then centrifuged for 10 min at 14,000 g at 4 °C. The VFA profiles (acetic, propionic, and butyric) were determined using a gas chromatograph (7890A GC, Agilent Technologies, Santa Clara, CA, USA) described by^45^. The remaining supernatant sample was centrifuged (10,000; 10 min) to separate microbial cells and the supernatant containing the enzyme. The1 precipitate was tested for microbial protein and the supernatant was tested for enzyme activity such as amylase, carboxymethyl cellulase, β-glucosidase^46^, and protease^47^.

### In vitro rumen digestibility

The in vitro rumen digestibility analysis was conducted using the two-stage in-vitro method^42^. Five hundred milligrams of substrate were weighed and placed in 100 mL serum bottle, which was then incubated at 39 °C for 48 h. After 48 h of incubation, the substrate samples were filtered to separate from rumen fluid. The substrates were used analysis of in vitro rumen DM digestibility (IVDMD), in vitro OM digestibility (IVOMD), in vitro CP digestibility (IVCPD), and in vitro CF digestibility (IVCFD). In the second stage, the other samples were added with 6 mL of 20% HCL and 2 mL pepsin solution. The samples were incubated at 39 °C for 48 h. At the end of incubation, the sample substrate was filtered to separate from rumen fluid and was analyzed for IVDMD, IVOMD, IVCPD, and IVCFD. The digestibility of DM, OM, CP and CF was determined using the the following formula:

$${\text{DMD}}=\frac{{\text{DM}} \, \mathrm{ weight} \, \mathrm{ of } \, \mathrm{ the} \, \mathrm{ sample}-(\mathrm{DM } \, {\text{contained}} \, \mathrm{ in } \, {\text{residue}}-{\text{blank}})}{{\text{DM}} \, \mathrm{ weight} \, \mathrm{ of} \, \mathrm{ the} \, \mathrm{ sample}}$$

$${\text{OMD}}=\frac{{\text{OM}} \, \mathrm{ weight} \, \mathrm{ of} \, \mathrm{ the} \, \mathrm{ sample}-(\mathrm{OM } \, \mathrm{contained } \, \mathrm{in } \, {\text{residue}}-{\text{blank}})}{\mathrm{OM } \, {\text{weight}} \, \mathrm{ of} \, \mathrm{ the} \, \mathrm{ sample}}$$

$${\text{CPD}}=\frac{\mathrm{CP } \, \mathrm{weight } \, \mathrm{of } \, \mathrm{the sample}-(\mathrm{CP } \, {\text{contained}} \, \mathrm{ in } \, {\text{residue}}-{\text{blank}})}{\mathrm{CP } \, \mathrm{weight } \, \mathrm{of } \, {\text{the}} \, \mathrm{ sample}}$$

$${\text{CFD}}=\frac{{\text{CF}} \, \mathrm{ weight} \, \mathrm{ of} \, \mathrm{ the} \, \mathrm{ sample}-(\mathrm{CF } \, {\text{contained}} \, \mathrm{ in} \, \mathrm{ residue}-{\text{blank}})}{\mathrm{CF } \, \mathrm{weight } \, {\text{of}} \, \mathrm{ the} \, \mathrm{ sample}}$$

### Statistical analysis

All treatments are replicated three times and collected data includes pH, NH_3_, total VFA, acetic, propionic, butyric, ratio of acetate and butyrate, microbial protein, protozoa cells, methane, amylase, carboxymethyl cellulase, β-glucosidase, protease, IVDMD, IVOMD, IVCPD, and IVCFD. The experiment data were analyzed by one-way ANOVA. If there is a significant difference in values (*P* < 0.05) were continued with Duncan's new multiple range test (DMRT) to find out the difference in the mean value between each treatment. Data analysis uses the Statistical Product and Service Solution (SPSS) 16 application (IBM, USA) (Supplementary Tables). 

## Conclusions

The use of 200 µL/L essential oil of nutmeg (*Myristica fragrans* Houtt.) in vitro was found to be the optimal concentration for reducing methane production without negatively affecting rumen fermentation, total nutrient digestibility (organic matter, dry matter, and crude fiber), and the activities of rumen enzymes (amylase, carboxymethyl cellulase, and β-Glucosidase). Furthermore, the in vitro addition of essential oil reduced protease enzyme activity and crude protein digestibility at 48 h incubation, representing digestibility in the rumen. Total crude protein digestibility improved at 96 h of incubation, indicating enhanced digestion in the post-rumen and optimal nutrition for the host animal.

### Supplementary Information

Supplementary Tables.

## Data Availability

The authors confirm that the data supporting the findings of this study are available within the article and its supplementary materials.

## References

[1] Soliva CR, Amelchanka SL, Duval SM, Kreuzer M (2011). Ruminal methane inhibition potential of various pure compounds in comparison with garlic oil as determined with a rumen simulation technique (Rusitec). Br. J. Nutr..

[2] Vaghar Seyedin SM, Zeidi A, Chamanehpour E, Nasri MHF, Vargas-Bello-Pérez E (2022). Methane emission: Strategies to reduce global warming in relation to animal husbandry units with emphasis on ruminants. Sustainability (Switzerland).

[3] Reisinger A (2021). How necessary and feasible are reductions of methane emissions from livestock to support stringent temperature goals?. Philos. Trans. R. Soc. A Math. Phys. Eng. Sci..

[4] Tapio I, Snelling TJ, Strozzi F, Wallace RJ (2017). The ruminal microbiome associated with methane emissions from ruminant livestock. J. Anim. Sci. Biotechnol..

[5] Morgavi DP, Kelly WJ, Janssen PH, Attwood GT (2013). Rumen microbial (meta)genomics and its application to ruminant production. Animal.

[6] Islam M, Lee SS (2019). Advanced estimation and mitigation strategies: A cumulative approach to enteric methane abatement from ruminants. J. Anim. Sci. Technol..

[7] Benchaar C (2020). Feeding oregano oil and its main component carvacrol does not affect ruminal fermentation, nutrient utilization, methane emissions, milk production, or milk fatty acid composition of dairy cows. J. Dairy Sci..

[8] Giorgino A, Federica R, Emanuela V, Domenico B, Damiano C, Marta G, Valentina B, Giorgia B, Stefania B, Francesca C, Lucrezia D, Gilberto M, Richard P, Victor S, Claudio V (2023). Effect of dietary organic acids and botanicals on metabolic status and milk parameters in mid-late lactating goats. Animals.

[9] Wierup M (2001). The Swedish experience of the 1986 year ban of antimicrobial growth promoters, with special reference to animal health, disease prevention, productivity, and usage of antimicrobials. Microb. Drug Resist..

[10] Cieslak A, Szumacher-Strabel M, Stochmal A, Oleszek W (2013). Plant components with specific activities against rumen methanogens. Animal.

[11] Ashokkumar K, Simal-Gandara J, Murugan M, Dhanya MK, Pandian A (2022). Nutmeg (*Myristica*
*fragrans* Houtt.) essential oil: A review on its composition, biological, and pharmacological activities. Phytother. Res..

[12] Belgacem D, Ali K, Rachid R, Djazia A (2016). Effect of Pituranthos scoparius essential oils on reducing methanogenesis in cheep. In vitro study. Indian J..

[13] Nooriyan Soroor, M. E. & Rouzbehan, Y. Effect of essential oils of eucalyptus (Eucalyptus globulus Labill) and Angelica (Heracleum persicum Desf. ex Fischer) on in vitro ruminal fermentation, protozoal population and methane emission using Afshari Sheep Inoculum. *J. Agr. Sci. Tech* **19** (2017).

[14] Patra AK, Yu Z (2012). Effects of essential oils on methane production and fermentation by, and abundance and diversity of, rumen microbial populations. Appl. Environ. Microbiol..

[15] Kamra DN, Agarwal N, Chaudhary LC (2006). Inhibition of ruminal methanogenesis by tropical plants containing secondary compounds. Int. Congr. Ser..

[16] Günal M, Pinski B, AbuGhazaleh AA (2017). Evaluating the effects of essential oils on methane production and fermentation under in vitro conditions. Ital. J. Anim. Sci..

[17] Khateri N, Azizi O, Jahani-Azizabadi H (2017). Effects of a specific blend of essential oils on apparent nutrient digestion, rumen fermentation and rumen microbial populations in sheep fed a 50:50 alfalfa hay:Concentrate diet. Asian-Australas J. Anim. Sci..

[18] Zurak D, Kristina K, Aladrović J (2023). Metabolism and utilisation of non-protein nitrogen compounds in ruminants: A review. J. Cent. Eur. Agric..

[19] Busquet M, Calsamiglia S, Ferret A, Kamel C (2005). Screening for effects of plant extracts and active compounds of plants on dairy cattle rumen microbial fermentation in a continuous culture system. Anim. Feed Sci. Technol..

[20] Cobellis G (2015). Evaluation of the effects of mitigation on methane and ammonia production by using *Origanum*
*vulgare* L. and *Rosmarinus*
*officinalis* L. essential oils on in vitro rumen fermentation systems. Sustainability (Switzerland).

[21] Burt S (2004). Essential oils: Their antibacterial properties and potential applications in foods—A review. Int. J. Food Microbiol..

[22] Diether NE, Willing BP (2019). Microbial fermentation of dietary protein: An important factor in diet–microbe–host interaction. Microorganisms.

[23] Zhou R (2020). Effects of oregano essential oil on in vitro ruminal fermentation, methane production, and ruminal microbial community. J. Dairy Sci..

[24] Wang B, Jia M, Fang L, Jiang L, Li Y (2018). Effects of eucalyptus oil and anise oil supplementation on rumen fermentation characteristics, methane emission, and digestibility in sheep. J. Anim. Sci..

[25] Karlsson J, Ramin M, Kass M, Lindberg M, Holtenius K (2019). Effects of replacing wheat starch with glycerol on methane emissions, milk production, and feed efficiency in dairy cows fed grass silage-based diets. J. Dairy Sci..

[26] Adriani A, Asra R, Novianti S, Fatati F (2019). The effect of *Coleus*
*amboinicus* L. supplementation on in vitro digestibility. Pak. J. Nutr..

[27] Duval SM, McEwan NR, Graham RC, Wallace RJ, Newbold CJ (2007). Effect of a blend of essential oil compounds on the colonization of starch-rich substrates by bacteria in the rumen. J. Appl. Microbiol..

[28] Wallace RJ, McEwan NR, McIntosh FM, Teferedegne B, Newbold CJ (2002). Natural products as manipulators of rumen fermentation. Asian-Australas J. Anim. Sci..

[29] Kala A, Kamra DN, Agarwal N, Chaudhary LC (2017). Effect of a blend of essential oils on buffalo rumen microbial and enzyme profiles and in vitro feed fermentation. Anim. Nutr. Feed Technol..

[30] Zhang H, Lang X, Li X, Chen G, Wang C (2022). Effect of *Zanthoxylum bungeanum* essential oil on rumen enzyme activity, microbiome, and metabolites in lambs. PLoS One.

[31] Wink, M. & Schimmer, O. Molecular Modes of Action of Defensive Secondary Metabolites. Functions and Biotechnology of Plant Secondary Metabolites: Second Edition vol. 39 (2010).

[32] Tager LR, Krause KM (2011). Effects of essential oils on rumen fermentation, milk production, and feeding behavior in lactating dairy cows. J. Dairy Sci..

[33] Hubi Zulfa I, Bachruddin Z, Kurniawati A (2019). Effects of lemongrass leaves as essential oil sources on rumen microbial ecology and nutrient digestibility in an in vitro system. Pak. J. Nutr..

[34] Hart KJ, Yáñez-Ruiz DR, Duval SM, McEwan NR, Newbold CJ (2008). Plant extracts to manipulate rumen fermentation. Anim. Feed Sci. Technol..

[35] Rosochacki SJ (2005). Skeletal muscle and liver protein degradation in mice divergently selected for low and high body weight over 108 generations. Arch. Anim. Breed..

[36] McIntosh FM (2003). Effects of essential oils on ruminal microorganisms and their protein metabolism. Appl. Environ. Microbiol..

[37] Wang HT, Hsu JT (2005). Optimal protease production condition for *Prevotella ruminicola* and characterization of its extracellular crude protease. Anaerobe.

[38] Xiao J (2011). Interaction of dietary polyphenols with bovine milk proteins: Molecular structure-affinity relationship and influencing bioactivity aspects. Mol. Nutr. Food Res..

[39] Kurade NP, Jaitak V, Kaul VK, Sharma OP (2010). Chemical composition and antibacterial activity of essential oils of *Lantana camara*, *Ageratum houstonianum* and *Eupatorium adenophorum*. Pharm. Biol..

[40] AOAC. Official Methods of Analysis. *AOAC International* (2005).

[41] Menke KH (1979). The estimation of the digestibility and metabolizable energy content of ruminant feedingstuffs from the gas production when they are incubated with rumen liquor in vitro. J. Agric. Sci..

[42] Tilley JMA, Terry RA (1963). A two-stage technique for the in vitro digestion of forage crops. Grass Forage Sci..

[43] Abreu A (2004). Effects of *Sapindus saponaria* fruits on ruminal fermentation and duodenal nitrogen flow of sheep fed a tropical grass diet with and without legume. J. Anim. Sci..

[44] Broderick GA, Kang JH (1980). Automated simultaneous determination of ammonia and total amino acids in ruminal fluid and in vitro media. J. Dairy Sci..

[45] Erwin ES, Marco GJ, Emery EM (1961). Volatile fatty acid analyses of blood and rumen fluid by gas chromatography. J. Dairy Sci..

[46] Halliwell G (1961). The action of cellulolytic enzymes from *Myrothecium verrucaria*. Biochem. J..

[47] Bergmeyer, H. in *Methods of Enzymatic Analysis*. Verlag Chemie, Weinheim.

